# Physical activity during early life and the risk of all-cause mortality in midlife: findings from a birth cohort study

**DOI:** 10.1093/eurpub/ckad084

**Published:** 2023-06-28

**Authors:** Natan Feter, Jayne S Leite, Marina K Weymar, Samuel C Dumith, Daniel Umpierre, Eduardo L Caputo

**Affiliations:** Postgraduate Program in Epidemiology, School of Medicine, Universidade Federal do Rio Grande do Sul, Porto Alegre, Rio Grande do Sul, Brazil; Postgraduate Program in Health Sciences, School of Medicine, Universidade Federal do Rio Grande do Sul, Porto Alegre, Rio Grande do Sul, Brazil; Postgraduate Program in Physical Education, School of Physical Education, Universidade Federal de Pelotas, Pelotas, Rio Grande do Sul, Brazil; Postgraduate Program in Health Sciences, School of Medicine, Universidade Federal do Rio Grande, Rio Grande, Rio Grande do Sul, Brazil; Postgraduate Program in Health Sciences, School of Medicine, Universidade Federal do Rio Grande do Sul, Porto Alegre, Rio Grande do Sul, Brazil; Postgraduate Program in Physical Education, School of Physical Education, Universidade Federal de Pelotas, Pelotas, Rio Grande do Sul, Brazil

## Abstract

**Background:**

The objective of this study was to examine the association between physical activity during childhood and adolescence and the risk of all-cause mortality in midlife. We analyzed data from a birth cohort (The 1958 National Child Development Survey), including births in England, Wales and Scotland.

**Methods:**

Physical activity was assessed using questionnaires at ages 7, 11 and 16. Death certificates defined all-cause mortality. Cumulative exposure, sensitive and critical periods, and physical activity trajectory from childhood to adolescence were tested using multivariate Cox proportional hazard models. The sweep the death was confirmed was defined as the time event.

**Results:**

From age 23 to 55, 8.9% of participants (*n* = 9398) died. Physical activity in childhood and adolescence affected the risk of all-cause mortality in midlife. In men, physical activity at ages 11 [hazard ratio (HR): 0.77; 95% confidence interval (CI): 0.60–0.98] and 16 (HR: 0.60; 95% CI: 0.46–0.78) was associated with reduced risk of all-cause mortality. In women, physical activity at age 16 (HR: 0.68; 95% CI: 0.48–0.95) was associated with reduced risk of all-cause mortality. Physical activity in adolescence eliminated the risk of all-cause mortality associated with physical inactivity in adulthood in women.

**Conclusions:**

Physical activity during childhood and adolescence was associated with reduced risk of all-cause mortality with different effects by sex.

## Introduction

Physical activity benefits health in all life stages, including childhood and adolescence. However, many school-aged children and adolescents do not engage in recommended levels of physical activity per week.^1^ This finding is significant considering that physical activity levels in youth are likely to persist during the adolescence–adulthood transition.^2^^,^^3^ A previous cohort study with 4257 adolescents showed that objectively measured physical activity, exceptionally light intensity, decreases between 12 and 16 years while sedentary time increases in the same period.^3^

Consequently, numerous large cohort studies have investigated the long-term consequences of physical activity at early ages.^3–9^ For example, active boys and girls aged between 12 and 16 have fewer depressive symptoms in early adulthood^3^ and improved executive function at age 50^7^ than their inactive counterparts. However, such effects may differ by sex. Active males at 16 gained body mass index (BMI) faster than inactive peers in the adolescence–adulthood transition, while active females had slower BMI gain in the same period.^6^ Similarly, executive function at age 50 may be improved by different levels of physical activity at age 16 in men; however, this positive association was observed only in women with the highest physical activity level at age 16.^7^

Numerous studies have confirmed the association between physical inactivity and the risk of all-cause and specific-cause mortality.^10–12^ Previous estimates showed that 1 in 10 premature deaths worldwide in 2008 was caused by physical inactivity.^13^ While the protective effect of physical activity on the risk of all-cause mortality is well established, the evidence regarding the relationship between physical activity in early life stages and mortality is limited. Previous studies have not found an association between adolescent physical activity and all-cause and cardiovascular mortality in adulthood.^14^^,^^15^ However, methodological characteristics, primarily retrospective design, might have biased these findings.

Even though most studies have reported a positive effect of physical activity in childhood and adolescence in several health indicators during adulthood, it is still scantly explored whether and to which extent physical activity in these life stages may attenuate the risk of premature all-cause mortality. Therefore, the aim of this study was to identify (i) the best life course model to explain the association between physical activity in childhood and adolescence and the risk of premature all-cause mortality and (ii) the risk of premature all-cause mortality in midlife by physical activity during childhood and adolescence. Based on the previous literature, we hypothesized that physical activity in childhood and adolescence is associated with a reduced risk of premature all-cause mortality. Furthermore, analyses were stratified by sex, considering the sex differences in the impact of physical activity on cardiovascular health.^16^

## Methods

We used data from the 1958 National Child Development Study (NCDS), a population-based, ongoing cohort study with individuals born in a specific week of March 1958 in England, Scotland and Wales (*N* = 18 555). A full description of the cohort is available elsewhere.^17^ The 1958 cohort was approved by the National Health Service Research Ethics committee, and written informed consent was given by the parents of study participants before the start of data collection. The manuscript is in accordance with the guidelines for STrengthening the Reporting of OBservational studies in Epidemiology (STROBE).^18^

### All-cause mortality

Deaths were ascertained systematically by the Centre for Longitudinal Studies upon receipt of death certificates from the National Health Service Central Register (NHSCR). It includes all deaths up to December 2013. We could not retrieve information on the exact death date and cause as the database with such information was under a special license when the data for this study were analyzed. Therefore, the sweep that death was confirmed was determined as the age of death.^19^ Information on death was obtained from the NHSCR, including individuals lost to follow-up in the cohort. The follow-up time was calculated from birth to the date of death for participants and up to December 2013 for survivors.

### Physical activity

A face-to-face interview-administered paper-based questionnaire assessed physical activity at ages 7, 11 and 16. Variables were operationalized based on previous NCDS publications, as reported in Supplementary table S1.^20^ At age 7, physical activity was considered as a mother's perception of a child's activity compared with the activity of children of the same age (inactive, normally active and overactive). Children who were rated as normally active or overactive were considered physically active. Questions about the frequency of playing games or sports outdoors and indoors, dancing and swimming were used to generate a composite score for ages 11 and 16, as previously recommended.^20^ Scores were categorized into quartiles, with participants in the three highest quartiles classified as physically active. Adulthood physical activity was created based on the physical activity level of each participant in the last wave before he or she was recorded as dead or the latest physical activity level if the participant was censored.

### Possible covariates

All multivariate analyses were adjusted for the following variables: sex, breastfeeding, low birth weight, mother’s socioeconomic status (SES), maternal smoking during pregnancy, chronic conditions, and smoking and alcohol intake at age 16. Sex, breastfeeding, low birth weight, mother’s SES, maternal smoking during pregnancy and chronic conditions were collected at the birth sweep. The mother’s SES was defined as the husband’s occupation. Occupations were categorized into four social class groups: professional/managerial (classes I and II), unskilled nonmanual (class III nonmanual), skilled manual (class III manual) and unskilled manual (classes IV and V; this category included cases in which there was no male head of household).^21^ Maternal smoking was defined as none, sometimes, medium and heavy. Chronic conditions included asthma, heart conditions, epilepsy and diabetes diagnosed at ages 7, 11 and 16. Smoking at age 16 was categorized as no smoking, less than one pack per week, one pack per week or more. Alcohol intake at age 16 was defined as currently drinking or not.

### Statistical analysis

Analyses were conducted to ensure no violation of normality, linearity, multicollinearity and homoscedasticity assumptions. First, a chi-squared test for heterogeneity was adopted to assess possible group differences. Second, three distinct Cox proportional hazard regression models were used to test the longitudinal association between physical activity in childhood and adolescence and the risk of all-cause mortality in midlife. Model 1 was performed without adjusting for any covariates (crude analysis). Model 2 included the early life characteristics of the individuals. Model 3 included the previous model plus behaviors during age 16. Model 4 included the previous model plus adulthood physical activity. Proportional hazard assumptions were assessed using global proportional hazards and plotting Schoenfeld residuals against time.

Third, we investigated the best model to explain the association between physical activity during early life and the risk of all-cause mortality in midlife. Four hypotheses were drawn based on the structural modeling for binary exposure variables: cumulative, sensitive, critical and pathways.^22^^,^^23^ Cumulative exposure suggests that the exposure affects the outcome at all time points similarly. The sensitive period suggests that the exposure affects the outcome at all time points with different magnitudes. A critical period indicates that exposure affects the outcome at only one time point. The pathway hypothesis suggests that the intensity of exposure that affects the outcome depends on the later exposure. Each nested model (cumulative, sensitive and critical hypotheses) was tested against the model of the pathway hypothesis using the log-likelihood ratio test. When nested models provided a similar fit to the pathway model (*P* > 0.05), the one with the lowest Akaike information criterion (AIC) was selected. Finally, Nelson–Aalen curves were plotted against the best-fit physical activity model to illustrate the cumulative hazard of risk of all-cause mortality by physical activity level. Considering the sex differences in the impact of physical activity on cardiovascular health, analyses were stratified by sex.^16^ Exploratory stratified analysis was used to investigate the influence of adulthood physical activity in the association between PA in childhood and adolescence and the risk of all-cause mortality.

## Results

Of the 18 555 participants enrolled in the 1958 NCDS, 9398 had data on all variables of interest for the present study (Supplementary figure S1). As shown in Supplementary table S2, participants whose mothers were in low socioeconomic positions and smoked heavily during pregnancy and those who smoked at age 16 were more likely to die before age 55 than their respective counterparts. Between ages 23 and 55, a significant difference in the incidence rate (IR) of premature all-cause mortality between men [IR: 5.5; 95% confidence interval (CI): 4.8–6.2] and women (IR: 3.5; 95% CI: 3.0–4.1) was observed (*P* < 0.001). Men were more likely to have chronic conditions, drink alcoholic beverages and be smokers at age 16 than women (Supplementary table S3). No difference was observed in physical activity at age 7 between the sexes. However, men were more physically active at ages 11 and 16. As shown in Supplementary table S4, participants who were included in the present study were more likely to drink alcoholic beverages and be smokers and physically inactive at age 16 compared with those who were excluded. No other meaningful differences were observed between groups.

Table 1 illustrates the best model estimation for physical activity and the risk of premature all-cause mortality. In men, accumulation and sensitivity hypotheses provided a similar explanation to the most complex, saturated model. However, the sensitive model had the lowest AIC value. In women, the sensitive model was the only model with non-significant *P* values. Therefore, the sensitive model best explained the association between physical activity in childhood and adolescence and the risk of premature all-cause mortality in both men and women.

**Table 1:** Best model estimation for physical activity and the risk of all-cause mortality

	LL	AIC	*P* value
Men			
No effect	−2826.31	5672.63	<0.001
Critical period			
Age 7 years	−2808.12	5638.24	<0.001
Age 11 years	−2688.04	5398.09	<0.001
Age 16 years	−2816.98	5655.96	<0.001
Accumulation	−2665.71	5353.42	0.190
Sensitive period	−**2663.60**	**5353.19**	**0.343**
Saturated model	−2661.35	5356.70	
Women			
No effect	−1725.73	3471.46	<0.001
Critical period			
Age 7 years	−1715.17	3452.33	<0.001
Age 11 years	−1603.91	3229.82	<0.001
Age 16 years	−1724.21	3470.43	<0.001
Accumulation	−1594.38	3210.76	0.044
Sensitive period	−**1591.38**	**3208.76**	**0.138**
Saturated model	−1587.90	3209.79	

Bold numbers indicate the model that best explained the association between physical activity in childhood and adolescence and premature all-cause mortality. LL, Log-likelihood ratio test.

Table 2 shows the summary IRs and HRs for the sex-stratified association between physical activity during childhood and adolescence and premature all-cause mortality. The IR was 27% and 31% lower in active than in inactive boys at ages 11 and 16. Among women, active participants at age 16 had an IR of all-cause mortality 23% lower than inactive peers. In men, physical activity at ages 11 and 16 was associated with 23% and 40% decreases in risk of premature all-cause mortality. The magnitude of the effect was similar among the adjusted models, although slight differences in HR were observed in model 4. In women, physical activity at age 16 was associated with a 32% decrease in the risk of premature all-cause mortality. However, the association became statistically non-significant in the final adjusted model. Therefore, stratified analysis was performed to investigate the association between PA in adulthood and adolescence with the risk of all-cause mortality.

**Table 2:** Physical activity at childhood and adolescence and the risk of all-cause mortality in midlife stratified by sex

	IR (95% CI)	Model 1	Model 2	Model 3	Model 4
Men					
Physical activity at age 7					
Inactive	6.60 (4.04–10.77)	1.00	1.00	1.00	1.00
Active	6.43 (5.84–7.08)	1.08 (0.61–1.92)	1.14 (0.62–2.08)	1.11 (0.61–2.04)	1.19 (0.58–2.41)
Physical activity at age 11					
Inactive	8.30 (7.13–9.67)	1.00	1.00	1.00	1.00
Active	6.02 (5.38–6.74)*	0.75 (0.60–0.94)	0.77 (0.61–0.99)	0.77 (0.60–0.98)	0.68 (0.51–0.90)
Physical activity at age 16					
Inactive	8.11 (6.91–9.51)	1.00	1.00	1.00	1.00
Active	5.59 (4.96–6.29)*	0.70 (0.56–0.88)	0.71 (0.56–0.90)	0.60 (0.46–0.78)	0.64 (0.46–0.88)
Women					
Physical activity at age 7					
Inactive	2.69 (1.28–5.65)	1.00	1.00	1.00	1.00
Active	4.19 (3.70–4.74)	1.68 (0.69–4.09)	1.67 (0.69–4.07)	1.65 (0.68–4.02)	1.41 (0.58–3.46)
Physical activity at age 11					
Inactive	4.61 (3.79–5.60)	1.00	1.00	1.00	1.00
Active	3.84 (3.30–4.46)	0.98 (0.72–1.33)	1.10 (0.80–1.52)	1.11 (0.81–1.52)	1.03 (0.71–1.48)
Physical activity at age 16					
Inactive	4.74 (3.85–5.84)	1.00	1.00	1.00	1.00
Active	3.65 (3.13–4.25)*	0.77 (0.57–1.03)	0.77 (0.57–1.05)	0.68 (0.48–0.95)	0.73 (0.50–1.08)

Model 1: crude analysis.

Model 2: adjusted for sex, breastfeeding, social class, low birth weight, maternal smoking during pregnancy, chronic conditions.

Model 3: model 2 plus smoking and alcohol drinking at age 16.

Model 4: model 3 plus physical activity in adulthood.

* *P* < 0.05 compared with inactive category.

**Figure 1:**
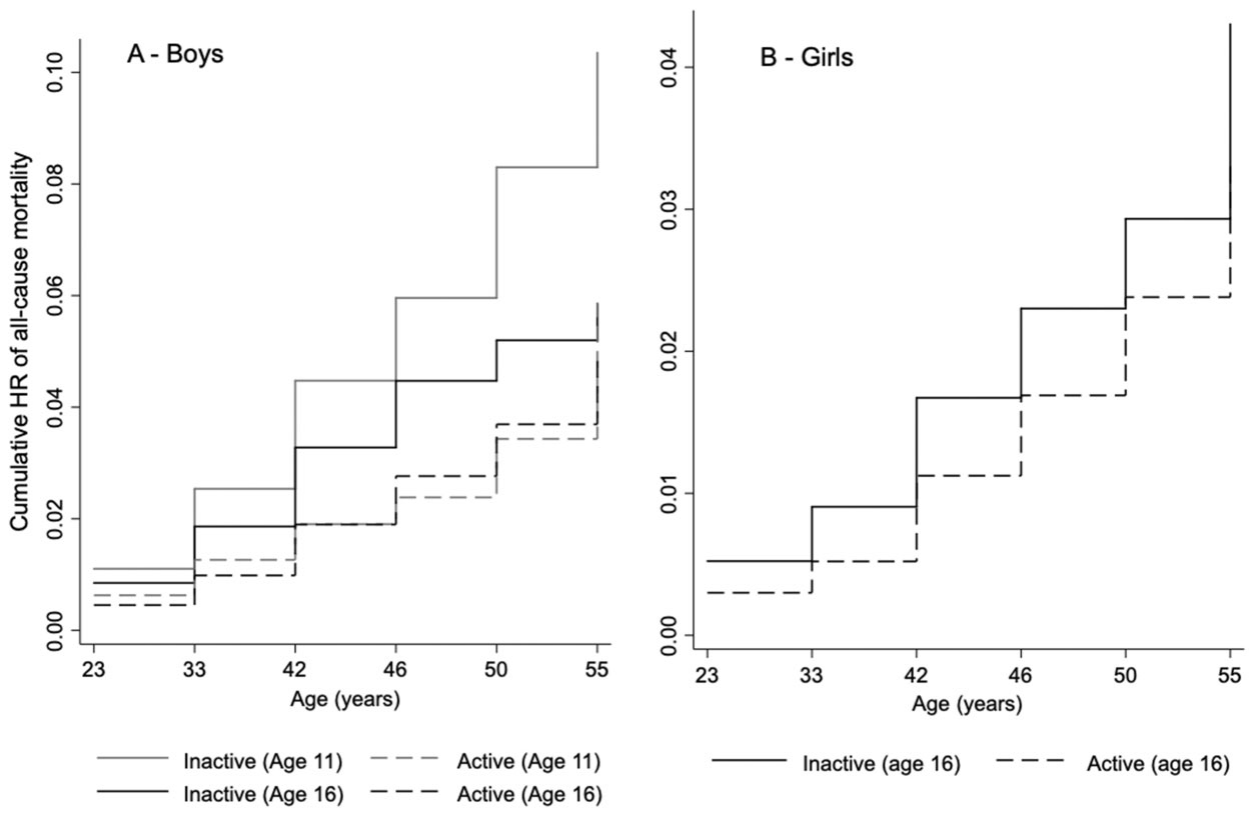
Nelson–Aalen cumulative hazard function on the association between physical activity at childhood and adolescence and the risk of all-cause mortality at midlife. (A) Boys and (B) girls

Figure 1 reported data on PA at ages 11 and 16 in men and 16 in women, as only PA at these ages was significantly associated with premature all-cause mortality. In men, the cumulative hazard for premature all-cause mortality is higher in individuals who were physically inactive at ages 11 and 16 than in peers who were active at the same ages. Additionally, inactive men at age 11 had the highest cumulative HR for premature all-cause mortality.

**Figure 2:**
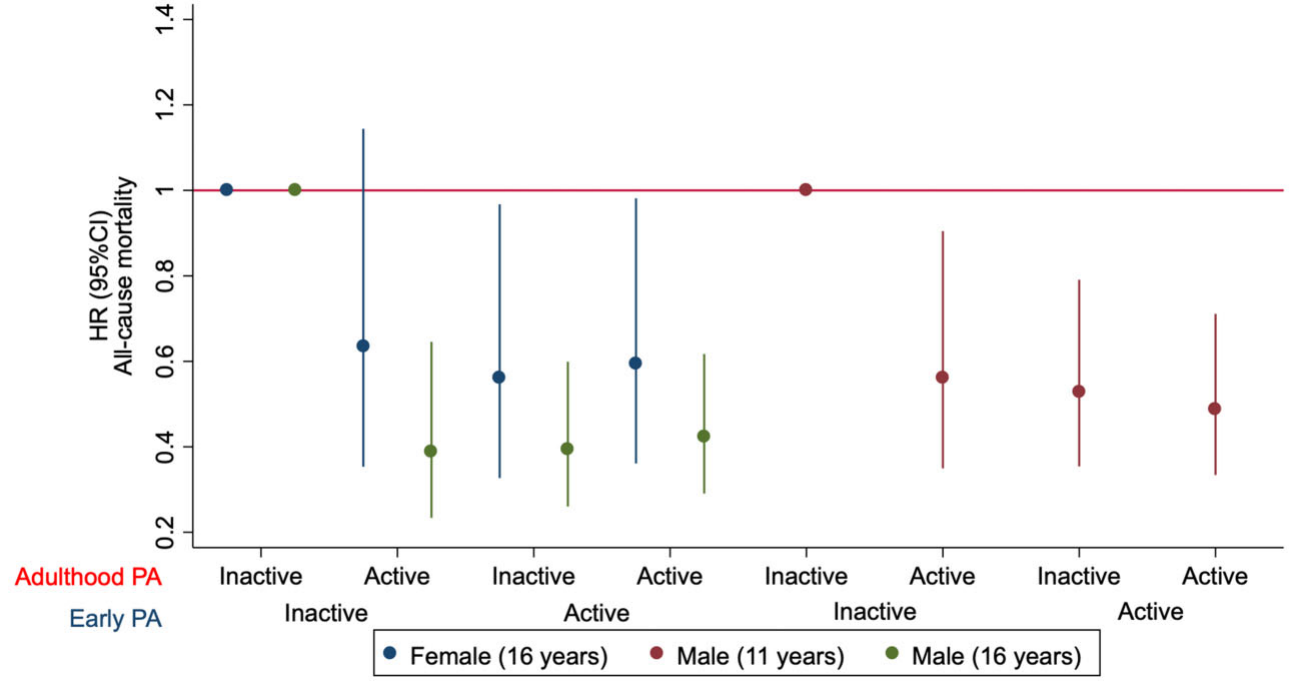
Association between physical activity at childhood and adolescence and the risk of all-cause mortality at midlife stratified by physical activity during adulthood. Adulthood PA: latest PA level recorded during follow-up. Early PA: childhood (age 11) and adolescence (age 16)

Stratified analysis showed that the beneficial association between childhood and adolescent PA with the risk of all-cause mortality was independent of adulthood PA in both sexes (figure 2). Physical activity in adulthood was associated with a reduced risk of mortality in men who were physically inactive at ages 11 [hazard ratio (HR): 0.56; 95% CI: 0.35–0.90) and 16 (HR: 0.39; 95% CI: 0.23–0.64). Physical activity in adulthood was not associated with a reduced risk of premature mortality in women who were physically inactive at age 16. On the other hand, physical activity at age 16 was associated with a reduced risk of all-cause mortality in women who were physically inactive in adulthood (HR: 0.56; 95% CI: 0.33–0.97). Similarly, physical activity at age 11 (HR: 0.53; 95% CI: 0.35–0.79) and 16 (HR: 0.39; 95% CI: 0.26–0.60) was associated with reduced mortality risk in men who were physically inactive in adulthood.

## Discussion

To our knowledge, this study is the first to use the life course approach to investigate the prospective association between physical activity in childhood and adolescence and the risk of premature all-cause mortality. Among men, physical activity in early and late adolescence significantly impacted the risk of premature all-cause mortality. Active boys aged 11 and 16 had a 23% and 40% lower risk of dying at age 55 or earlier, respectively. In women, those who were physically active in late adolescence had a reduced risk of premature all-cause mortality. Physical activity at age 16 was associated with a reduced risk of premature all-cause mortality in women.

Physical activity in adolescence was associated with all-cause mortality in both men and women, although an age difference was observed. In our sample, we observed a higher IR of premature all-cause mortality in men than in women, in consonance with previous studies.^24^ Physical activity has been associated with physical fitness in adolescents, including those aged 11 and 16.^25^^,^^26^ Indeed, boys with persistent high physical activity levels from age 11–16 showed superior cardiovascular fitness than peers who remained physically inactive in the same period.^25^ However, in a previous study, no association was observed between tracking physical activity from childhood to adolescence and physical fitness.^25^ Finally, evidence has established the dose–response relationship between physical fitness and the risk of all-cause mortality.^27^ In adolescents, cardiorespiratory fitness levels are associated with several health indicators, including adiposity, cardiovascular and skeletal health, depression, anxiety, and academic performance.^28^ Considering previous evidence, we hypothesized that, for men, the higher risk of premature all-cause mortality might be reduced by improved or preserved physical fitness through an active childhood–adolescence transition. Health promotion policies and physical activity programs should be designed to stimulate a healthy and physically active lifestyle from childhood.

Evidence suggests that the prevalence of non-communicable chronic diseases (NCD) (e.g. type 2 diabetes,^29^ hypertension^30^) among adolescents is increasing worldwide. In 2019, NCD accounted for roughly 40% of all adolescent deaths.^31^ Although the known protective effects of physical activity on such conditions, the prevalence of physical inactivity is persistently higher in adolescents than in adults.^32^ A study with 1.6 million adolescents from 146 countries showed that four in five adolescents were physically inactive in 2016, with a higher prevalence among girls (84.7%) than among boys (77.6%). Physical inactivity in early life stages has been linked to poor health status at older ages, including poor cognitive performance^7^ and increased risk of multimorbidity.^4^ In the present study, we showed that physical inactivity in adolescence was associated with a higher risk of all-cause mortality in women, even in those who were active in adulthood. This long-term effect from physical activity, also known as ‘physical activity legacy’, may be reinforced by future strategies to promote physical activity among children and adolescents.

In this sense, schools may play an essential role in physical activity promotion for school-going children and adolescents.^33^ However, the effects of a school-based intervention to promote physical activity are inconsistent. A previous meta-analysis pooled data from 18 805 children and 16 435 adolescents to show that interventions focusing only on education, environment or policies have inconclusive evidence of physical activity promotion.^34^ However, multicomponent strategies that embraced the entire scholar community (school plus community or family) had strong evidence of improving physical activity in children and adolescents. Also, lack of social support, including parental support, is an important barrier to physical activity in this population.^35^ Similarly, previous evidence has suggested a bidirectional association between physical activity and mental health.^36^ In addition, adults with depression are more likely to remain inactive throughout the adulthood.^37^ Physical activity is a complex, multifactorial behavior that needs consistent surveillance and promotion. In addition, hazardous health behaviors including physical inactivity, smoking, and substance abuse are likely to be presented in clusters, reinforcing the need for health education and promotion strategies. Community or family-tailored physical activity programs may be an efficient strategy for children and adolescents. Physical activity cannot be seen only as an exhaustive daily obligation, especially for children. The 2020 WHO guidelines on physical activity and sedentary behavior suggest that every move counts.^38^ Replacing small doses of sedentary behavior with physical activity may improve several health indicators, including the risk of all-cause mortality.^39^ Since 1958, when this survey started, we have children and adolescents spending more time in sedentary activities, including video games, smartphones and computers.^40^ The ‘physical activity legacy’ concept may help reinforce the importance of physical activity in children and adolescents to reduce the burden and mortality attributable mainly to NCD in older ages.

This study has numerous limitations that need to be highlighted. First, physical activity was assessed using questionnaires. Objective measures of physical activity, such as pedometers and accelerometers, were not available for large-scale, population-based studies such as the NCDS in 1958. In addition, information regarding the intensity and duration of LTPA was not assessed. Although this limits the capacity for a recommendation, the types of PA covered in the questionnaire are everyday activities adolescents perform. Second, we could not determine each participant's exact date and cause of death. The 1958 NCDS dataset with this information (SN 7717) was available at the UK Data Service under a special license and only to researchers in the European Economic Area. Third, reverse causality may not be ruled out due to the study design. However, only 46 (9.2%) of all deaths were reported at the age 23 sweep. Our findings were also confirmed by sensitive analysis, excluding deaths registered during this sweep. Fourth, participants who died during follow-up were more likely to be men, had low weight at birth, and were smokers at age 16, so residual confounding cannot be ruled out. Finally, attrition bias may not be completely ruled out, as the frequency of hazardous health behaviors was higher in included than in excluded participants. However, our study assessed physical activity prospectively, not retrospectively, as in the available literature.^14^^,^^15^ This is a strength of our study regarding the reliability of physical activity data.

In conclusion, physical activity during adolescence was associated with a reduced risk of premature all-cause mortality during midlife. Although our results need to be confirmed by studies with physical activity measured by objective assessments (i.e. accelerometers), future public health policies and physical activity programs, especially for school-going youths, may reinforce the importance of physical activity at young ages to reduce the risk of mortality in later life.

## Supplementary Material

ckad084_Supplementary_Data

## Data Availability

The datasets generated and/or analyzed during the current study are available in the Data Archive at the University of Essex, https://beta.ukdataservice.ac.uk/datacatalogue/studies/study?id=5560. The STATA commands used during the present study are publicly available at https://osf.io/m7xup/. Physical activity in childhood and adolescence was associated with a lower risk of premature all-cause mortality. Women who were physically active at age 16 showed a lower risk of early mortality regardless of the physical activity level in adulthood. Physical activity in childhood was associated with a reduced risk of all-cause mortality in men.
