# Supplementary material for: Physical activity during early life and the risk of all-cause mortality in midlife: findings from a birth cohort study
Source: Eur J Public Health. 2023 Jun 28;33(5):872–7. doi: 10.1093/eurpub/ckad084 (PMC10567256; doi:10.1093/eurpub/ckad084)
Supplement: ckad084_Supplementary_Data [file ckad084_supplementary_data.pdf]

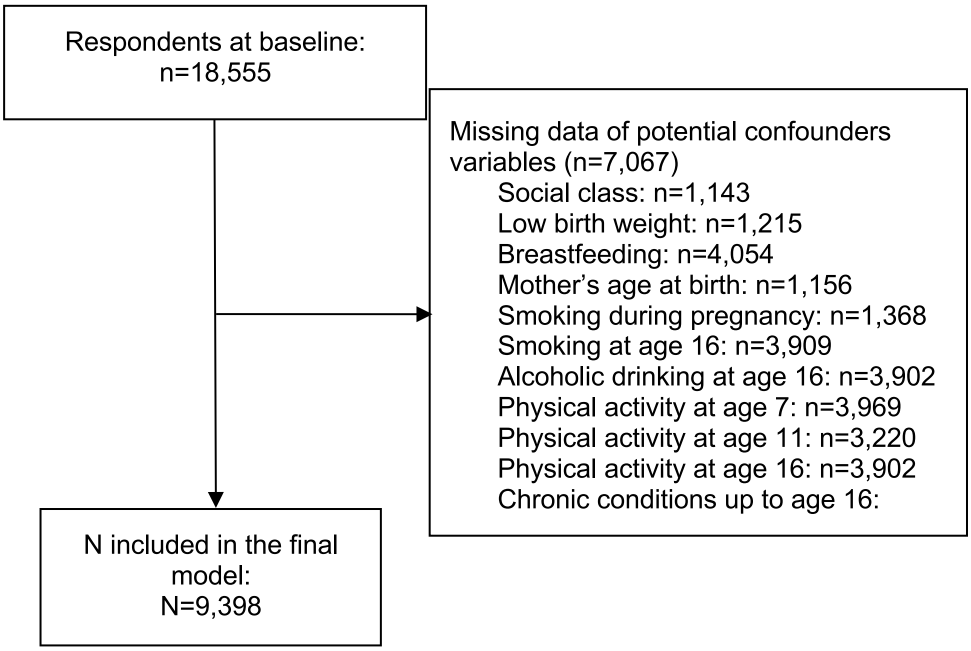

Supplementary Figure 1. Study workflow.

Supplementary Table S1. Assessment and operationalization of physical activity in the present study.

Sweep	Responder	Question/Instrument	Categories	
			NCDS	Present study
Age 7 sweep	Mother	Question: Is the child normally active, inactive and quiet (prefer to sit and watch), or restless and overactive (can't keep still)?	Inactive; Normally; active; Over active	Inactive; Normally active + Over active
Age 11 sweep	Mother	Excluding holidays away from home how often has your child used the following in his/her spare time in the past 12 months?	Never or hardly ever; sometimes; Often (nearly every day)	Often was scored as 2, sometimes scored as 1, never and not available were scored as 0. The scores were summed across the five variables and the resulting 11 categories collapsed to 4. The three most active categories were merged as active.
Age 16 sweep	Individual	Below is a list of four things which many people do in their spare time. Please show by ringing one of the numbers for each one whether this is something that you do often, sometimes, never or hardly never.	Never or hardly ever; sometimes; Often	Often was scored as 2, sometimes scored as 1, never and not available were scored as 0. The scores were summed across the five variables and the resulting 11 categories collapsed to 4. The three most active categories were merged as active.
Age 23 sweep	Individual	Please tell me from this card, how often you have done each one over the past 4 weeks.	Not at all in the last 4 weeks; once in the last 4 weeks; 2 or 3 times in the last 4 weeks; once or twice per week; 3 or 4 times per week; 5 times per week	Inactive (less than once per week); active (once per week or more)
Age 33 sweep	Individual	How often do you take part in any activity of this type?	Less often; 2-3 times per month; once a week; 2-3 days a week; 4-5 days a week; every day or most days	Inactive: less than once a week; Active: once a week or more
Age 42 sweep	Individual	How often do you take part in any activity of this type?	Less often; 2-3 times per month; once a week; 2-3 days a week; 4-5 days a week; every day	Inactive: less than once a week; Active: once a week or more
Age 50 sweep	Individual	How often do you take part in any activity of this type?	Less often; 2-3 times per month; once a week; 2-3 days a week; 4-5 days a week; every day	Inactive: less than once a week; Active: once a week or more

Age 55 sweep	Individual	How often do you take part in any activity of this type?	Less often; 2-3 times per month; once a week; 2-3 days a week; 4-5 days a week; every day	Inactive: less than once a week; Active: once a week or more

NCDS: National Child Development Survey.

Supplementary Table S2. Characteristics of the included participants. 1958 National Child Development Study. N=18,555

	Died (n=1,660)	Not died (n=16,895)	p-value
Sex, n (%)			<0.001
Male	975 (10.2)	8,618 (89.8)	
Female	685 (7.6)	8,277 (92.4)	
Breastfeed (n=14,495), n (%)			0.015
No	248 (5.4)	4,345 (94.6)	
Yes	443 (4.5)	9,459 (95.5)	
Low birth weight (n=17,338), n (%)			<0.001
No	1,162 (7.2)	14,949 (92.8)	
Yes	439 (35.8)	788 (64.2)	
Social class (n=17,412), n (%)			<0.001
No male head at household	107 (11.2)	851 (88.8)	
I - professional	38 (5.1)	708 (94.9)	
II - managerial and technical/intermediate	155 (7.3)	1,978 (92.7)	
III - skilled, non-manual	122 (7.7)	1,469 (92.3)	
III - skilled, manual	815 (9.7)	7,559 (90.3)	
IV - partly skilled	176 (8.8)	1,818 (91.2)	
V - unskilled	201 (12.4)	1,415 (87.6)	
Mother age at birth (n=17,398), mean years (SD)	27.5 (6.0)	27.5 (5.7)	0.640
Mother smoking during pregnancy (n=17,187), n (%)			<0.001
None	942 (8.3)	10,463 (91.7)	
Sometimes	100 (10.3)	867 (89.7)	
Medium	303 (11.2)	2,403 (88.8)	
Heavy	243 (11.5)	1,866 (88.5)	
Alcohol drinking at age 16 (n=14,650), n (%)			<0.001
No	206 (6.0)	3,230 (94.0)	
Yes	467 (4.2)	10,747 (95.8)	
Smoking at age 16 (n=14,650), n (%)			<0.001
No smoker	442 (4.3)	9,927 (95.7)	
Less than 1 pack per week	69 (4.3)	1,540 (95.7)	
1 pack per week or more	162 (6.1)	2,510 (93.9)	
Chronic conditions up to age 16* (n=18,532), n (%)			<0.001
No	1,511 (9.3)	14,805 (90.7)	
Yes	141 (6.4)	2,071 (93.6)	
Physical activity at age 7 (n=14,583), n (%)			0.221
Inactive	23 (4.1)	543 (95.9)	
Normally active	551 (4.6)	11,342 (95.4)	
Over active	115 (5.4)	2,009 (94.6)	
Physical activity at age 11 (n=15,332), n (%)			0.011
Least active	266 (5.6)	4,448 (94.4)	
2nd	178 (4.2)	4,057 (95.8)	
3rd	186 (4.7)	3,791 (95.3)	
Most active	108 (4.8)	2,298 (95.5)	
Physical activity at age 16 (14,650), n (%)			0.001
Least active	239 (5.7)	3,954 (94.3)	
2nd	172 (4.1)	3,989 (95.9)	
3rd	168 (4.2)	3,873 (95.8)	
Most active	94 (4.6)	2,161 (95.8)	

* Chronic conditions: asthma, heart conditions, epilepsy and diabetes at ages 7, 11 and 16.

Supplementary Table S3. Characteristics of the included participants stratified by sex. 1958 National Child Development Study. N=9,398.

	Male (n=4,764)	Female (n=4,634)	p-value
Breastfeed, n (%)			0.215
No	1,508 (31.6)	1,412 (30.5)	
Yes	3,256 (68.4)	3,222 (69.5)	
Low birth weight, n (%)			<0.001
No	4,571 (95.9)	4,374 (94.4)	
Yes	193 (4.1)	260 (5.6)	
Social class, n (%)			0.885
No male head at household	201 (4.1)	205 (4.4)	
I - professional	194 (4.1)	196 (4.2)	
II - managerial and technical/intermediate	620 (13.0)	612 (13.2)	
III - skilled, non-manual	480 (10.1)	437 (9.4)	
III - skilled, manual	2,303 (48.3)	2,243 (48.4)	
IV - partly skilled	553 (11.6)	559 (12.1)	
V - unskilled	413 (8.7)	382 (8.2)	
Mother age at birth, mean years (SD)	27.5 (5.6)	27.6 (5.7)	0.653
Mother smoking during pregnancy, n (%)			0.666
None	3,210 (67.4)	3,115 (67.2)	
Sometimes	299 (6.3)	266 (5.7)	
Medium	715 (15.0)	711 (15.3)	
Heavy	540 (11.3)	542 (11.7)	
Alcohol drinking at age 16, n (%)			<0.001
No	199 (4.2)	336 (7.3)	
Yes	4,565 (95.8)	4,298 (92.7)	
Smoking at age 16, n (%)			<0.001
No smoker	2,964 (62.2)	3,017 (65.1)	
Less than 1 pack per week	575 (12.1)	716 (15.5)	
1 pack per week or more	1,225 (25.7)	90 (19.4)	
Chronic conditions up to age 16*, n (%)			<0.001
No	3,966 (83.2)	4,062 (87.7)	
Yes	798 (16.8)	572 (12.3)	
Physical activity at age 7, n (%)			<0.001
Inactive	177 (3.7)	189 (4.1)	
Normally active	3,829 (80.4)	3,850 (83.1)	
Over active	758 (15.9)	595 (12.8)	
Physical activity at age 11, n (%)			<0.001
Least active	1,177 (24.7)	1,359 (29.3)	
2nd	1,328 (27.9)	1,419 (30.6)	
3rd	1,324 (27.8)	1,267 (27.3)	
Most active	935 (19.6)	589 (12.7)	
Physical activity at age 16, n (%)			<0.001
Least active	531 (11.1)	681 (14.7)	
2nd	1,449 (30.4)	1,796 (38.8)	
3rd	1,636 (35.6)	1,494 (32.2)	
Most active	1,088 (22.8)	663 (14.3)	

Supplementary Table S4. Characteristics of the overall participants. 1958 National Child Development Study. N=18,555

	Excluded (n=9,160)	Included (n=9,398)	p-value
Sex, n (%)			0.005
Male	4830 (52.8%)	4764 (50.7%)	
Female	4323 (47.2%)	4634 (49.3%)	
Breastfeed, n (%)			0.030
No	1673 (32.8%)	2920 (31.1%)	
Yes	3424 (67.2%)	6478 (68.9%)	
Low birth weight, n (%)			<0.001
No	7166 (90.3%)	8945 (95.2%)	
Yes	774 (9.7%)	453 (4.8%)	
Social class, n (%)			<0.001
No male head at household	552 (6.9%)	406 (4.3%)	
I - professional	356 (4.4%)	390 (4.1%)	
II - managerial and technical/intermediate	901 (11.2%)	1232 (13.1%)	
III - skilled, non-manual	675 (8.4%)	917 (9.8%)	
III - skilled, manual	3829 (47.8%)	4546 (48.4%)	
IV - partly skilled	883 (11.0%)	1112 (11.8%)	
V - unskilled	821 (10.2%)	795 (8.5%)	
Mother age at birth, mean years (SD)	27.37 (5.85)	27.53 (5.62)	0.052
Mother smoking during pregnancy, n (%)			<0.001
None	5083 (65.2%)	6325 (67.3%)	
Sometimes	402 (5.2%)	565 (6.0%)	
Medium	1280 (16.4%)	1426 (15.2%)	
Heavy	1027 (13.2%)	1082 (11.5%)	
Alcohol drinking at age 16, n (%)			<0.001
No	4,191 (79.8%)	5,160 (54,9%)	
Yes	1,064 (20.2%)	4,238 (45.1%)	
Smoking at age 16, n (%)			<0.001
No smoker	4390 (83.5%)	5981 (63.6%)	
Less than 1 pack per week	318 (6.1%)	1291 (13.7%)	
1 pack per week or more	547 (10.4%)	2126 (22.6%)	
Chronic conditions up to age 16*, n (%)			<0.001
No	8292 (90.8%)	8028 (85.4%)	
Yes	842 (9.2%)	1370 (14.6%)	
Physical activity at age 7, n (%)			0.74
Inactive	200 (3.9%)	366 (3.9%)	
Normally active	4214 (81.3%)	7679 (81.7%)	
Over active	771 (14.9%)	1353 (14.4%)	
Physical activity at age 11, n (%)			<0.001
Least active	2180 (36.7%)	2536 (27.0%)	
2nd	1488 (25.1%)	2747 (29.2%)	
3rd	1387 (23.4%)	2591 (27.6%)	
Most active	882 (14.9%)	1524 (16.2%)	
Physical activity at age 16, n (%)			<0.001
Least active	2981 (56.8%)	1212 (12.9%)	
2nd	916 (17.4%)	3245 (34.5%)	
3rd	851 (16.2%)	3190 (33.9%)	
Most active	504 (9.6%)	1751 (18.6%)	

* Chronic conditions: asthma, heart conditions, epilepsy and diabetes at ages 7, 11 and 16.

STROBE Statement—Checklist of items that should be included in reports of ***cohort studies***

	Item No	Recommendation	Included
**Title and abstract**	1	(*a*) Indicate the study's design with a commonly used term in the title or the abstract<br>(*b*) Provide in the abstract an informative and balanced summary of what was done and what was found	✓<br>✓
**Introduction**			
Background/rationale	2	Explain the scientific background and rationale for the investigation being reported	✓
Objectives	3	State specific objectives, including any prespecified hypotheses	✓
**Methods**			
Study design	4	Present key elements of study design early in the paper	✓
Setting	5	Describe the setting, locations, and relevant dates, including periods of recruitment, exposure, follow-up, and data collection	✓
Participants	6	(*a*) Give the eligibility criteria, and the sources and methods of selection of participants. Describe methods of follow-up<br>(*b*) For matched studies, give matching criteria and number of exposed and unexposed	✓
Variables	7	Clearly define all outcomes, exposures, predictors, potential confounders, and effect modifiers. Give diagnostic criteria, if applicable	✓
Data sources/ measurement	8*	For each variable of interest, give sources of data and details of methods of assessment (measurement). Describe comparability of assessment methods if there is more than one group	✓
Bias	9	Describe any efforts to address potential sources of bias	✓
Study size	10	Explain how the study size was arrived at	✓
Quantitative variables	11	Explain how quantitative variables were handled in the analyses. If applicable, describe which groupings were chosen and why	✓
Statistical methods	12	(*a*) Describe all statistical methods, including those used to control for confounding<br>(*b*) Describe any methods used to examine subgroups and interactions<br>(*c*) Explain how missing data were addressed<br>(*d*) If applicable, explain how loss to follow-up was addressed<br>(*e*) Describe any sensitivity analyses	✓
**Results**			
Participants	13*	(a) Report numbers of individuals at each stage of study—eg numbers potentially eligible, examined for eligibility, confirmed eligible, included in the study, completing follow-up, and analysed<br>(b) Give reasons for non-participation at each stage<br>(c) Consider use of a flow diagram	✓
Descriptive data	14*	(a) Give characteristics of study participants (eg demographic, clinical, social) and information on exposures and potential confounders<br>(b) Indicate number of participants with missing data for each variable of interest<br>(c) Summarise follow-up time (eg, average and total amount)	✓
Outcome data	15*	Report numbers of outcome events or summary measures over time	✓

Main results	16	(*a*) Give unadjusted estimates and, if applicable, confounder-adjusted estimates and their precision (eg, 95% confidence interval). Make clear which confounders were adjusted for and why they were included<br>(*b*) Report category boundaries when continuous variables were categorized<br>(*c*) If relevant, consider translating estimates of relative risk into absolute risk for a meaningful time period	✓
Other analyses	17	Report other analyses done—eg analyses of subgroups and interactions, and sensitivity analyses	✓
**Discussion**			
Key results	18	Summarise key results with reference to study objectives	✓
Limitations	19	Discuss limitations of the study, taking into account sources of potential bias or imprecision. Discuss both direction and magnitude of any potential bias	✓
Interpretation	20	Give a cautious overall interpretation of results considering objectives, limitations, multiplicity of analyses, results from similar studies, and other relevant evidence	✓
Generalisability	21	Discuss the generalisability (external validity) of the study results	✓
**Other information**			
Funding	22	Give the source of funding and the role of the funders for the present study and, if applicable, for the original study on which the present article is based	N/A

*Give information separately for exposed and unexposed groups.

Note: An Explanation and Elaboration article discusses each checklist item and gives methodological background and published examples of transparent reporting. The STROBE checklist is best used in conjunction with this article (freely available on the Web sites of PLoS Medicine at http://www.plosmedicine.org/, Annals of Internal Medicine at http://www.annals.org/, and Epidemiology at http://www.epidem.com/). Information on the STROBE Initiative is available at http://www.strobe-statement.org.
